# Neutrophil percentage to albumin ratio is associated with stroke-associated pneumonia and poor outcome in patients with spontaneous intracerebral hemorrhage

**DOI:** 10.3389/fimmu.2023.1173718

**Published:** 2023-06-14

**Authors:** Xin-Ni Lv, Yi-Qing Shen, Zuo-Qiao Li, Lan Deng, Zi-Jie Wang, Jing Cheng, Xiao Hu, Ming-Jun Pu, Wen-Song Yang, Peng Xie, Qi Li

**Affiliations:** ^1^ Department of Neurology, The First Affiliated Hospital of Chongqing Medical University, Chongqing, China; ^2^ National Health Commission (NHC) Key Laboratory of Diagnosis and Treatment on Brain Functional Diseases, The First Affiliated Hospital of Chongqing Medical University, Chongqing, China; ^3^ Department of Neurology and Neurosurgery, The Third Affiliated Hospital of Chongqing Medical University, Chongqing, China; ^4^ Department of Neurology, The Second Affiliated Hospital of Anhui Medical University, Hefei, Anhui, China

**Keywords:** intracerebral hemorrhage, pneumonia, inflammation, neutrophil percentage-to-albumin ratio, outcome

## Abstract

**Background:**

The purpose of this study was to investigate the diagnostic performance of the neutrophil percentage-to-albumin ratio (NPAR) for predicting stroke-associated pneumonia (SAP) and functional outcome in patients with intracerebral hemorrhage (ICH).

**Methods:**

We analyzed our prospective database of consecutive ICH patients who were admitted to the First Affiliated Hospital of Chongqing Medical University from January 2016 to September 2021. We included subjects with a baseline computed tomography available and a complete NPAR count performed within 6h of onset. The patients’ demographic and radiological characteristics were analyzed. Good outcome was defined as a modifed Rankin Scale score of 0-3 at 90 days. Poor outcome was defined as a modifed Rankin Scale score of 4-6 at 90 days. Multivariable logistic regression models were used to investigate the association between NPAR, SAP, and functional outcome. Receiver operating characteristic (ROC) curve analysis was conducted to identify the optimal cutoff of NPAR to discriminate between good and poor outcomes in ICH patients.

**Results:**

A total of 918 patients with ICH confirmed by non-contrast computed tomography were included. Of those, 316 (34.4%) had SAP, and 258 (28.1%) had poor outcomes. Multivariate regression analysis showed that higher NPAR on admission was an independent predictor of SAP (adjusted odds ratio: 2.45; 95% confidence interval, 1.56-3.84; P<0.001) and was associated with increased risk of poor outcome (adjusted odd ratio:1.72; 95% confidence interval, 1.03–2.90; P=0.040) in patients with ICH. In ROC analysis, an NPAR of 2 was identified as the optimal cutoff value to discriminate between good and poor functional outcomes.

**Conclusion:**

Higher NPAR is independently associated with SAP and poor functional outcome in patients with ICH. Our findings suggest that early prediction of SAP is feasible by using a simple biomarker NPAR.

## 1 Introduction

Spontaneous intracerebral hemorrhage (ICH) is one of the least treatable types of stroke with a high disability rate among survivors (1). It is estimated to account for 11%-30% of all forms of strokes and afflicts over 2 million deaths worldwide each year (1–3). Stroke-associated pneumonia (SAP) is a common and serious complication in patients with stroke and is associated with a fourfold increased risk of a poor outcome (4, 5). SAP can occur as a result of aspiration of oropharyngeal secretions or from a systemic inflammatory response to the stroke itself. The risk of SAP is highest in the first few days after stroke and may persist for several weeks (6). Several factors have been identified as predictors of poor outcomes after ICH, including age, baseline hematoma volume, hematoma growth (HG), presence of intraventricular hemorrhage (IVH), perihematomal edema (PHE) expansion, and Glasgow Coma Scale (7, 8). In addition, there is compelling evidence that inflammatory reactions may play a critical role in SAP, suggesting that clinical biomarkers of inflammation may be associated with poor outcome after ICH (9).

In previous studies, absolute neutrophils, as a major proportion of the leukocyte population, have been correlated with poor outcome after ICH (10–12). From a pathophysiological standpoint, oxidative stress and inflammation are key processes that can lead to further neuronal damage following intracerebral hemorrhage (ICH) (13–15). Neutrophils are the earliest leukocyte subtype to migrate to the central nervous system and initiate the inflammatory response in animal models (9, 16). In humans with ICH, a few neutrophils are typically found in the perihematomal region shortly after the onset of hemorrhage (within 8 hours) (17). These studies suggest that neutrophils may play a critical role in the inflammatory response during ICH. Several studies showed that lower levels of albumin on admission are associated with an increased risk of coronary disease and ICH mortality (18, 19).

The activation of systemic inflammatory responses following ICH is known to contribute to secondary injury and increase the risk of SAP (6). This immune response is triggered by cerebral damage and influenced by the sympathetic pathway and hypothalamus-pituitary-adrenal axis, leading to a prolonged inflammatory reaction that can decrease immune activity. Therefore, assessing the inflammatory status of ICH patients is a critical aspect of predicting and preventing secondary complications such as pneumonia (20). Given the increasing need to understand the relationship between inflammatory markers and SAP and clinical outcomes in the ICH population, the neutrophil percentage-to-albumin ratio (NPAR) has emerged as an easily available parameter that reflects the inflammatory response in patients with ICH. It is worth noting, however, that elevated NPAR levels may also be indicative of inflammation resulting from SAP. The purpose of our study was to determine whether the NPAR at the time of admission was associated with SAP and functional outcome after ICH.

## 2 Subjects and methods

### 2.1 Study design and population

We prospectively recruited spontaneous ICH patients hospitalized at the First Affiliated Hospital of Chongqing Medical University, Chongqing, China from January 2016 to September 2021 who underwent CT scan and admission routine blood examinations within 6 hours of admission. Written informed consent was obtained from all patients or their legal representatives. The study protocols were conducted in accordance with the Declaration of Helsinki. The Ethics Committee of the First Affiliated Hospital of Chongqing Medical University approved the study protocol. For the present analysis, we included patients with: 1) age >18 years, 2) diagnosis of acute spontaneous ICH, (3) baseline non-contrast CT (NCCT) images and serum neutrophils and albumin measurement availability, and (4) 3 months modified Rankin Scale (mRS) available. Subjects were excluded if: 1) primary IVH, 2) other types of ICH (trauma, tumor, neoplasia, cerebral aneurysm, vascular malformation, hemorrhagic transformation of acute ischemic stroke), 3) lost to follow-up or missing data on outcome, 4) completed surgical treatment (craniotomy or hematoma aspiration).

### 2.2 Clinical data collection and image analysis

The clinical and demographic data, including age, sex, admission systolic blood pressure (SBP) and diastolic blood pressure (DBP), Glasgow Coma Scale (GCS), the National Institutes of Health Stroke Scale (NIHSS), duration of hospital stay, history of hypertension, history of diabetes, as well as risk factors such as smoking, drinking were prospectively collected. At the time of hospitalization, 5 ml of venous blood was collected. All blood samples, including examinations of the leucocyte count, neutrophil count, lymphocyte count, and albumin were collected from each patient at admission in EDTA tubes (for plasma) or vacutainer tubes (for serum). The cell counts were measured by using an auto-analyzer. All biochemical parameters were analyzed using an automatic biochemical analyzer. The NPAR is defined as the neutrophil percentage divided by the number of albumin.

The modified Rankin Scale (mRS) is a commonly used clinical tool to assess the functional outcome of stroke patients in some clinical trials (21, 22). The mRS score ranges from 0 to 6, with 0 indicating no symptoms, 1 indicating no significant disability, and scores of 2 to 5 representing various degrees of disability, up to severe disability or death. The primary clinical outcome was a poor 3-month mRS score of 4-6 at follow-up. Three-month functional outcomes were assessed by trained telephone interviews with methodologic details described previously (22). Initial ICH and IVH volumes on computed tomography images were assessed with a semi-automated, computer-assisted planimetric measurement using previously described techniques (AnalyzeDirect medical imaging software, version 11.0; AnalyzeDirect, Inc.) (7). We outlined the hematoma and boundaries of perihematomal edema on axial slices in the region of interest in this software. A semiautomated threshold-based detection approach (range of 5-33 Hounsfield Units) was used to identify perihematomal edema regions (7). Hematoma location was classified as basal ganglia vs non-basal ganglia based on NCCT images. Additional images were obtained when the participant’s condition deteriorated or based on clinical need. All NCCT scans were independently evaluated by two trained neurologists. Both neurologists were blinded to clinical information.

### 2.3 Statistical analysis

Data analysis was performed using Statistical Package for the Social Sciences (SPSS) for Windows (Version 25.0; SPSS Inc., Chicago, IL, USA). Categorical variables were expressed as count (percentage), and continuous variables were presented as mean (± standard deviation [SD]) or median (interquartile range [IQR]). Comparisons were evaluated through the Mann-Whitney U-test, Student t-test, or Chi-square test as appropriate. The variables with P values <0.10 in univariate analysis and previously identified predictors of poor outcomes were entered into multivariate analysis. A multivariable logistic regression model was used to identify the risk factors and predictors and the results are expressed as odds ratios (OR) and 95% confidence interval (CI). In addition, we performed multivariate analyses by incorporating all independent variables into a stepwise forward logistic regression model to identify independent risk factors for 3-month poor outcomes after ICH. In this multivariate analysis, we adjusted for age, admission NIHSS score, time from onset to CT, basal ganglia location, SAP, baseline ICH volume, presence of IVH, and NPAR. In this model, multi-collinearity was estimated with the variance inflation factor (VIF). The variable we used for multivariate analysis was tolerance >0.1 and VIF <5. Receiver operating characteristic (ROC) curve analysis was performed to evaluate the predictive performance for poor functional outcomes by the NPAR values obtained at admission. The discrimination of NPAR was measured by the area under the curve (AUC). The optimal cut-off point of NPAR was determined by the Youden index. P values less than 0.05 were defined as statistically significant.

## 3 Results

### 3.1 Study population and clinical characteristics

A total of 918 patients were included in our final analysis. The baseline demographic, clinical, and radiological characteristics were listed in **Tables 1**, **2**. The study consisted of 636 men (69.3%) and 282 women (30.7%) with a mean age of 61.7 years (SD=13.9). A total of 258 (28.1%) patients had poor outcomes at 3 months after ICH. The median neutrophil percentage and NPAR were, respectively, 81.1 (IQR 73.2-87.7) and 2.0 (IQR 1.8-2.2). The mean of albumin was 40.5 g/L (SD=4.6).

**Table 1:** Comparison of baseline demographic, clinical, and radiological characteristics between Patients with and without poor outcome. (n=918).

Variable	Good outcome n=660 (71.9%)	Poor outcome n=258 (28.1%)	*P* Value
Mean age, years (SD)	60.2 (13.5)	65.5 (14.0)	<0.001
Sex, male, n (%)	462 (70.0)	174 (67.4)	0.450
Smoking, n (%)	321 (48.6)	98 (38.0)	0.005
Alcohol consumption, n (%)	232(35.2)	66 (25.6)	0.009
History of Hypertension, n (%)	464 (70.3)	179 (69.4)	0.772
History of Diabetes mellitus, n (%)	101 (15.3)	50 (19.4)	0.131
History of Ischemic stroke, n (%)	55 (8.3)	28 (10.9)	0.219
History of Haemorrhagic stroke, n (%)	52 (7.9)	28 (10.9)	0.147
Clinical features			
Admission SBP, mmHg (SD)	167.2 (28.4)	171.6 (32.4)	0.055
Admission DBP, mmHg (SD)	97.2 (18.0)	95.2 (18.5)	0.128
White blood cell count, 10^9^/L, Mean (SD)	8.7 (3.2)	10.6 (4.3)	<0.001
Hemoglobin, g/L, Mean (SD)	138.5 (17.8)	134.6 (19.3)	0.007
Median neutrophils percentage (IQR)	79.7 (71.6-86.6)	85.2 (77.9-90.0)	<0.001
Median NPAR (IQR)	1.9 (1.7-2.1)	2.1 (1.9-2.3)	<0.001
Median admission GCS score (IQR)	15.0 (14.0-15.0)	12.0 (6.0-14.0)	<0.001
Median admission NIHSS score (IQR)	5.0 (2.0-11.0)	15.0 (8.0-35.0)	<0.001
Median time from onset to imaging, h (IQR)	7.5 (2.6-23.4)	3.6 (1.7-11.8)	<0.001
Median hematoma volume, ml (IQR)	7.6 (3.1-15.2)	14.4 (7.1-35.9)	<0.001
IVH at baseline CT, n (%)	144 (21.8)	127 (49.2)	<0.001
Basal ganglia hematoma, n (%)	304 (46.1)	109 (42.2)	0.297
Pneumonia, n (%)	176 (26.7)	140 (54.3)	<0.001

ICH, Intracerebral hemorrhage; GCS, Glasgow Coma Scale; IVH, Intraventricular hemorrhage; IQR, Interquartile range; SD, Standard deviation; CT, Computed tomography; NIHSS, National Institute of health stroke scale; SBP, Systolic blood pressure; DBP, Diastolic blood pressure; IQR, inter-quartile range.

**Table 2:** Comparison of baseline demographic, clinical, and radiological characteristics between Patients with and without pneumonia. (n=918).

Variable	without Pneumonia n=602 (65.6%)	Pneumonia n=316 (34.4%)	*P* Value
Mean age, years (SD)	59.8 (13.6)	65.3 (13.6)	<0.001
Sex, male, n (%)	410 (68.1)	226 (71.7)	0.287
Smoking, n (%)	282 (46.8)	137 (43.4)	0.364
Alcohol consumption, n (%)	204 (33.9)	94 (29.7)	0.288
History of Hypertension, n (%)	410 (68.1)	233 (73.7)	0.065
History of Diabetes mellitus, n (%)	88 (14.6)	63 (19.9)	0.040
History of Ischemic stroke, n (%)	52 (8.6)	31 (9.8)	0.527
History of Haemorrhagic stroke, n (%)	44 (7.3)	36 (11.4)	0.034
Clinical features			
Admission SBP, mmHg (SD)	166.8 (29.1)	171.5 (30.5)	0.024
Admission DBP, mmHg (SD)	96.5 (17.8)	96.9 (18.9)	0.792
White blood cell count, 10^9^/L, Mean (SD)	8.7 (3.3)	10.3 (4.0)	<0.001
Hemoglobin, g/L, Mean (SD)	138.0 (17.9)	136.2 (19.6)	0.156
Median neutrophil percentage (IQR)	78.7 (70.2-86.6)	84.3 (79.0-89.1)	<0.001
Median NPAR (IQR)	1.9 (1.7-2.1)	2.1 (1.9-2.3)	<0.001
Median admission GCS score (IQR)	15.0 (14.0-15.0)	13.0 (9.0-14.0)	<0.001
Median admission NIHSS score (IQR)	5.0 (2.0-10.0)	13.0 (8.0-22.0)	<0.001
Median time from onset to imaging, h (IQR)	6.4 (2.7-22.5)	4.0 (1.7-22.2)	<0.001
Median hematoma volume, ml (IQR)	7.5 (2.7-15.2)	13.0 (6.5-26.0)	<0.001
IVH at baseline CT, n (%)	125 (20.8)	146 (46.2)	<0.001
Basal ganglia hematoma, n (%)	286 (47.5)	127(40.2)	0.034
Outcome			
In-hospital mortality, n (%)	28 (4.7)	33 (10.4)	0.001
90-day mortality, n (%)	67 (11.1)	80 (25.3)	<0.001
90-day mRS score, median (IQR)	1 (0-3)	3 (1-6)	<0.001

ICH, Intracerebral hemorrhage; GCS, Glasgow Coma Scale; IVH, Intraventricular hemorrhage; IQR, Interquartile range; SD, Standard deviation; CT, Computed tomography, NIHSS, National Institute of health stroke scale; SBP, Systolic blood pressure; DBP, Diastolic blood pressure; IQR, inter-quartile range; mRS, modified Rankin Scale.

**Figure 1:**
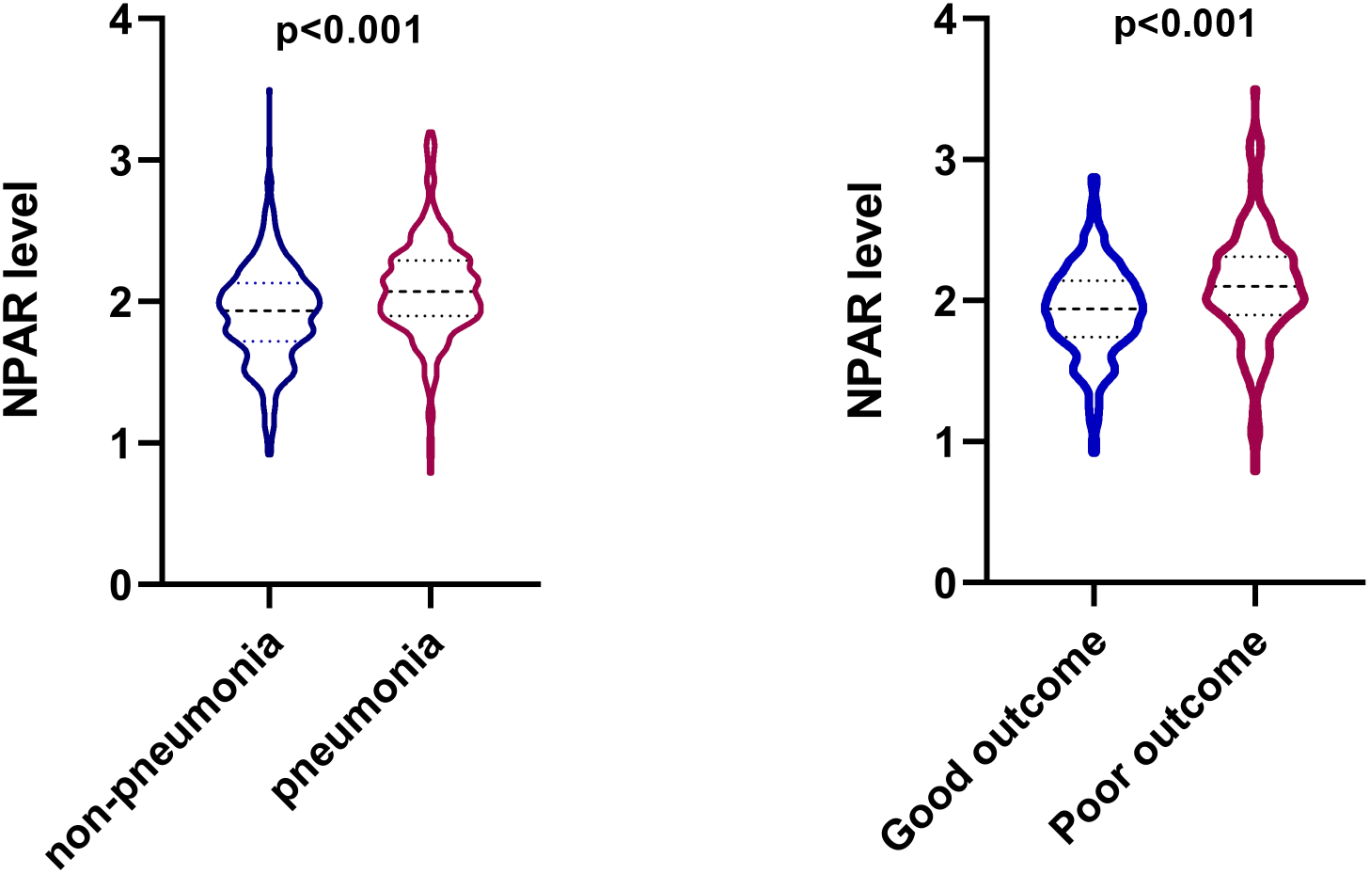
Violin plot that shows the dispersion of the neutrophil percentage-to-albumin ratio (NPAR) level in different groups in patients with intracerebral hemorrhage.

The clinical, laboratory, and imaging characteristics of ICH patients were illustrated in **Table 1**. Patients with poor outcomes had higher admission NIHSS scores (median [IQR] NIHSS score 15.0 [8.0-35.0] vs 5.0 [2.0-11.0]; P<0.001), lower GCS scores (median [IQR] GCS score 12.0 [6.0-14.0] vs 15.0 [14.0-15.0]; P<0.001), larger hematoma volume (median [IQR] volume 14.4 [7.1-35.9] vs 7.6 [3.1-15.2]; P<0.001), older age (mean [SD] age 65.5 [14.0] vs 60.2 [13.5]; P<0.001), and faster time from onset to imaging (median [IQR] time from onset to imaging 3.6 [1.7-11.8] vs 7.5 [2.6-23.4]; P<0.001) (**Table 1**). In terms of the laboratory values, patients with poor outcomes were more likely to have higher levels of white blood cell count (mean [SD] 10.6 [4.3] vs 8.7 [3.2]; P<0.001), neutrophil percentage (median [IQR] 85.2 [77.9-90.0] vs 79.7 [71.6-86.6]; P<0.001), and NPAR (median [IQR] 2.1 [1.9-2.3] vs 1.9 [1.7-2.1]; P<0.001) (**Table 1**; **Figure 1**). There were no differences in gender, history of hypertension as well as history of diabetes mellitus in those with and without poor outcomes.

Compared with patients in the pneumonia group, those in the non-pneumonia group were significantly younger (P<0.001) and had lower admission SBP (P=0.024), smaller baseline hematoma volume (P<0.001) and lower NIHSS score at admission (P<0.001). The SAP group had a significantly higher white blood cell count (mean [SD] 10.3 [4.0] vs 8.7 [3.3]; P<0.001), neutrophil percentage (median [IQR] 84.3 [79.0-89.1] vs 78.7 [70.2-86.6]; P<0.001) and NPAR (median [IQR] 2.1 [1.9-2.3] vs 1.9 [1.7-2.1]; P<0.001) than that of the non-SAP group (**Table 2**; **Figure 1**). Patients with stroke-associated pneumonia were more likely to have intraventricular hemorrhage (P<0.001), a history of diabetes mellitus (P=0.04), history of hemorrhagic stroke (P=0.034) and were associated with increased short-term mortality (P<0.001) as compared with those without pneumonia.

### 3.2 Multivariate analysis of predictors for SAP and poor outcome

**Figure 2:**
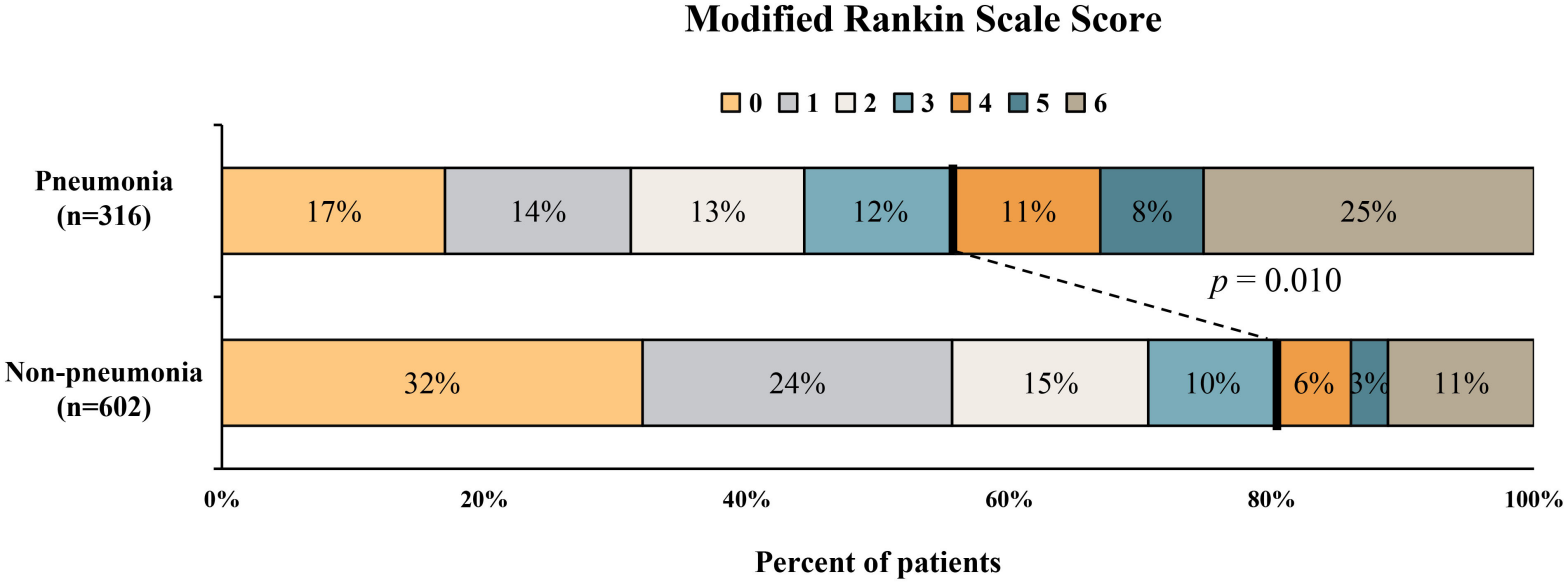
Distribution of modified Rankin Scale score in patients with and without pneumonia.

The distribution of 3-month mRS in patients with and without pneumonia is shown in **Figure 2**. Multivariable logistic regression analysis showed that NPAR was independently associated with the increased risk of pneumonia after adjusting for age, admission GCS score, baseline ICH volume, time from onset to CT imaging [adjusted odds ratio (aOR) = 2.45; 95% confidence interval (CI), 1.56-3.84; P< 0.001] (**Table 3**). As shown in **Table 4**, age (aOR = 1.03; 95% CI, 1.01-1.04; P< 0.001), admission NIHSS score (aOR = 1.08; 95% CI, 1.06-1.10; P< 0.001), baseline hematoma volume (aOR = 1.02; 95% CI, 1.01-1.03; P= 0.001), IVH at baseline (aOR = 1.55; 95% CI, 1.07-2.26; P=0.021), time from onset to CT (aOR = 0.99; 95% CI, 0.98-0.99; P=0.014), and the NPAR (aOR = 1.72; 95% CI, 1.03–2.90; P=0.040) were independent predictors of poor outcome after ICH. A ROC curve analysis was performed to obtain the optimal NPAR cut-off value for predicting poor outcomes after ICH. With a cut-off value of 2.0, NPAR showed a sensitivity of 72.1% and a specificity of 51.5%.

**Table 3:** Multivariate analysis of predictors for pneumonia.

Variable	Odds Ratio	95% Confidence Interval	P Value
Multivariate analysis			
Age, year	1.03	1.01-1.04	<0.001
Admission GCS score ^a^	0.82	0.78-0.87	<0.001
Baseline ICH volume, mL ^a^ Time from onset to CT, hours ^a^	1.00 1.00	0.99-1.01 0.99-1.01	0.941 0.518
NPAR	2.45	1.56-3.84	<0.001

ICH, Intracerebral hemorrhage; GCS, Glasgow Coma Scale; CT, Computed tomography; NPAR, neutrophil percentage-to-albumin ratio.

a Per unit change in regressor.

**Table 4:** Multivariate analysis of predictors for poor outcome (mRS 4-6) at 3 months.

Variable	Odds Ratio	95% Confidence Interval	P Value
Multivariate analysis ^a^			
Age, year	1.03	1.01-1.04	<0.001
Admission NIHSS score ^b^	1.08	1.06-1.10	<0.001
Baseline ICH volume, mL ^b^ IVH at baseline CT	1.02 1.55	1.01-1.03 1.07-2.26	0.001 0.021
Time from onset to CT, hour ^b^	0.99	0.98-0.99	0.014
NPAR	1.72	1.03-2.90	0.040

ICH, Intracerebral hemorrhage; GCS, Glasgow Coma Scale; IVH, Intraventricular hemorrhage; CT, Computed tomography; mRS, modified Rankin scale; NPAR, neutrophil percentage-to-albumin ratio.

a The analysis was adjusted for age, admission NIHSS score, time from onset to CT, basal ganglia location, SAP, baseline ICH volume, presence of IVH, and NPAR.

b Per unit change in regressor.

## 4 Discussion

In this study, a higher NPAR upon admission was identified as an independent and convenient biomarker of SAP and poor outcomes among ICH patients. Our findings suggest that NPAR, as a novel inflammatory biomarker, may have prognostic value and aid in risk stratification in patients with ICH.

Previous studies have shown that a higher level of neutrophils at admission is closely related to early neurological deterioration, in-hospital mortality, and poor outcomes at 3 months in patients with ICH (10). In addition to neutrophils, other inflammatory biomarkers, including lymphocytes, monocytes, and the neutrophil-lymphocyte ratio (NLR), have been studied in patients with ICH (10, 11, 23–26). Increasing evidence indicates that an elevated NLR is associated with hematoma enlargement, perihematomal edema growth, early neurological deterioration, and increased mortality at 3 months (10, 23–26). In our study, we found that patients with SAP had higher levels of neutrophils than those without, and the neutrophil-to-albumin ratio (NPAR) was associated with an increased risk of pneumonia after ICH. Our data suggest that early inflammatory responses may be detrimental to functional outcomes. Additionally, several studies have demonstrated that albumin is a suitable biomarker for predicting severe sepsis and mortality in patients with ICH (27, 28). An association between high albumin levels and better functional outcomes, as well as reduced mortality in acute ischemic stroke, suggests that albumin may have a neuroprotective effect (29). Prior study has recognized albumin as an important antioxidant component in plasma that participates in the delay phase of neuronal death (30). Decreased serum albumin may weaken the antioxidant effect and increase oxidative stress. Albumin and neutrophils both play critical roles in the inflammatory response during ICH. Furthermore, hypoalbuminemia is not uncommon in conditions such as malnutrition and renal or hepatic dysfunction, and is associated with poor overall health and frailty (31, 32). A study has reported that serum albumin levels can independently predict pneumonia in acute ischemic stroke patients (33), and early hypoalbuminemia is an independent factor for pneumonia and sepsis in ICH patients (34).

In combination, neutrophils and albumin may be more valuable and reliable biomarkers for predicting outcomes across various diseases, providing both nutritional and acute inflammatory information. The NPAR has been studied as an independent prognostic marker in patients with anti-N-methyl-D-aspartic acid receptor encephalitis, malignancy, and other diseases (35–37). One study reported that NPAR proved to be an independent predictor of short-term outcomes in acute ischemic stroke patients who received reperfusion therapy (37). Another study showed a positive correlation between higher NPAR on admission and the occurrence of ischemic stroke-associated infection (38). However, to date, no study has shown the association of NPAR with outcome in ICH patients. In our study of 918 ICH patients, we demonstrated that NPAR had good clinical value in predicting poor functional outcome. NPAR represents a reliable marker and dynamic index of systemic inflammation that integrates different factors of inflammation and immune response. It can integrate the likelihood of secondary cerebral injury and the susceptibility to post-stroke complications. Therefore, monitoring NPAR may assist clinicians in selecting patients at a higher risk of poor outcomes, especially in the emergency department. Our study not only evaluated the prognostic value of NPAR for SAP but also examined its prognostic value for poor outcomes in a large sample cohort, providing further insights into the complex relationship between acute inflammation and ICH.

Currently, the ICH score is the most commonly used prognostic tool for identifying patients with poor outcomes in clinical practice (39). Compared with the relatively complex clinical score, NPAR seems to be a simple and easily available biomarker for outcome stratification, as NPAR can be readily derived from routine blood test results. Future studies should focus on exploring the additional informative value of the NPAR trajectory over time to predict ICH outcomes. Additionally, the association between NPAR or other biochemical markers and clinical prognosis in specific subgroups, such as the severity of ICH or stratification by subtype of hemorrhage, should be investigated.

The present study has several noteworthy limitations. First, this is a single-center study with a limited sample size. Second, our results can be only applied to medically managed ICH patients due to the exclusion of patients who underwent surgical treatments. Third, our study only investigated the predictive value of NPAR on admission, the dynamic alteration of NPAR over time is still unknown. Future studies should focus on exploring the additional informative value of the NPAR trajectory over time to predict ICH outcome.

## 5 Conclusion

In the current study, the NAPR was independently associated with the increased risk of pneumonia and 3-month poor functional outcome. This ratio could be an accessible and appropriate serum biomarker to assist early decision-making in regard to risk stratification and management.

## Data availability statement

The original contributions presented in the study are included in the article/supplementary material. Further inquiries can be directed to the corresponding author.

## Ethics statement

The study was conducted in accordance with the Declaration of Helsinki and approved by the Ethics Committee of the First Affiliated Hospital of Chongqing Medical University (Approval No. 2017-075). The patients/participants provided their written informed consent to participate in this study.

## Author contributions

QL and X-NL: study concept and design. QL, X-NL, Y-QS, Z-QL, LD, Z-JW, JC, XH, M-JP, W-SY, and PX: acquisition of data. X-NL and Y-QS: statistical analysis. Analysis and interpretation of data: all authors. X-NL: drafting of the manuscript. QL, X-NL: critical revision of the manuscript for important intellectual content. QL, X-NL: obtained funding. QL: study supervision. All authors contributed to the article and approved the submitted version.
